# Inspiration From Games and Entertainment Artifacts: A Rising Paradigm for Designing Mechanisms and Algorithms in Robotics

**DOI:** 10.3389/frobt.2019.00003

**Published:** 2019-01-29

**Authors:** Ning Tan, Nishaan Brahmananthan, Rajesh Elara Mohan, Veerajagadheswar Prabakaran

**Affiliations:** ^1^School of Data and Computer Science, Sun Yat-sen University, Guangzhou, China; ^2^Key Laboratory of Machine Intelligence and Advanced Computing, Ministry of Education, Guangzhou, China; ^3^Engineering Product Development Pillar, Singapore University of Technology and Design, Singapore, Singapore

**Keywords:** design paradigm, robotic mechanisms, mobile robots, reconfigurable robotics, path planning

## Abstract

Games and toys have been serving as entertainment tools to humans for a long period of time. While except for entertainment, they can also trigger inspiration and enhance productivity in many other domains such as healthcare and general workplaces. The concept of the game is referred to a series of structured procedures (e.g., card games) and virtual programs. The entertainment artifacts could be a toy or even a handicraft, such as origami and kirigami, for entertainment purposes in a broader sense. Recently, the design of robots and relevant applications in robotics has been emerging in taking inspiration from Games and Entertainment Artifacts (GEA). However, there is a lack of systematic and general process for implementing a GEA-inspired design for developing robot-related applications. In this article, we put forward a design paradigm based on the inspiration of game and entertainment artifacts which is a systematic design approach. The design paradigm could follow two different processes which are driven by problems and solutions, respectively, using analogies of games and entertainment artifacts to build robotic solutions for solving real problems. The problem-driven process starts with an existing real-world problem, which follows the sequences of robotics problem search, robotics problem identification, GEA solution search, GEA solution identification, GEA principle extraction, and the principle implementation. Reversely, the solution-driven process follows the sequence of GEA solution search, GEA solution identification, GEA principle extraction, robotics problem search, robotics problem identification, and principle implementation. We demonstrate the application of the design paradigm using the case study of a new type of reconfigurable floor cleaning robot and its path planning algorithm.

## Introduction

**Figure 1:**
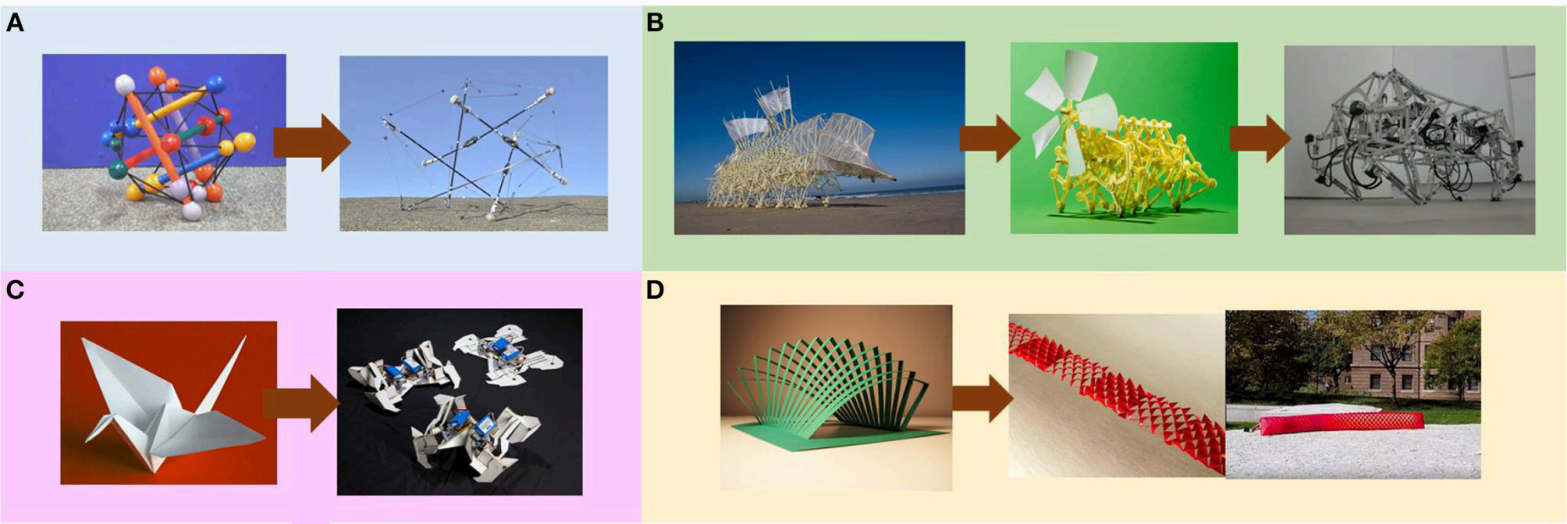
Examples of robots inspired by toys and handicraft. **(A)** SUPERball inspired by Skwish. **(B)** Quadruped robot inspired by a toy of Theo Jansen's strandbeest. **(C)** Robogami inspired by origami. **(D)** Robotic crawler inspired by kirigami.

Using analogies as design inspiration is intuitive and classical paradigm in robotics where the principle of an object in non-robotic domains is used to assist the design of robots or enhance the performance aspects in robotics. Similar to the counterparts in bio-inspired designs, games, and entertainment artifacts are an alternative design treasury for robotics. For example, as shown in Figure 1A, the the spherical underactuated planetary exploration robot ball (SUPERball) was a prototype developed by Dynamic Tensegrity Robotics Lab at NASA Ames Research Center, for the use of space exploration missions (Sabelhaus et al., 2015). The mechanical, structural design for the robot was inspired by a baby toy called Skwish. Landing of spacecraft on moons, planets, and asteroids without damaging them is a very complicated technological challenge faced by engineers and designers, and in general, most of the baby toys are also by default designed to be indestructible. The CareToy system was inspired by a commercially available gym for infants (Cecchi et al., 2016). The system is equipped with a variety of sensors, which can provide an intensive, individualized, home-based, and family-centered early-intervention program monitored by clinicians remotely. Likewise, origami (Figure 1C) is well known for its art of paper folding, which is often associated with Japanese culture. Origami can be seen as a way of creating low-cost toys for entertainment purposes. As a result, the robogami (i.e., origami-inspired robot) has been emerging in the market during the recent years, which enables low-cost and rapid prototyping of 3D robots from 2D sheets ranging in scale from the micrometer and millimeter range (Hawkes et al., 2010; Felton et al., 2014; Fitzner et al., 2017). Similarly, kirigami is a variation of origami that involves cutting of the paper, rather than solely folding the paper. The kirigami principle was harnessed to enhance the crawling capability of a soft actuator (Rafsanjani et al., 2018). The mechanical instabilities in the stretchable kirigami surfaces (Figure 1D) induced a transformation from flat sheets to 3D-textured surfaces akin to the scaled skin of snakes. The Theo Jansen mechanism (Figure 1B) is a planar linkage enabling mobility in five different motions including forward, backward, left turn, right turn and pivot turn with the use of just one actuator to drive the whole mechanism (García, 2015). One of our previously developed quadruped robots was inspired by a toy which was the scale model of the Theo Jansen's strandbeest (Nansai et al., 2013).

Even though there are a lot of works in bioinspiration and biomimetics (Bhushan, 2009; Sitti et al., 2013) ranging from rigid mechanisms (Zhou and Bi, 2012) to soft mechanisms (Kim et al., 2013) to algorithms (Zheng and Sicker, 2013), the bio-inspired design only has been considered in the paradigm or framework point of view in a short period of time. Over the past decade, the design paradigms of bio-inspired engineering design have been studied and presented in a number of articles (Vattam et al., 2007; Helms et al., 2009). Haberland et al. (Haberland and Kim, 2015) proposed the first general framework for determining bio-inspired relationships between design input variables and output objectives and constraints that are applicable to engineering systems. They used a case study of the relative running efficiency of rotary-kneed and telescoping-legged robots to demonstrate the application of the proposed framework.

Games and entertainment artifacts are also inspiring the design of mechanisms and algorithms in robotics. However, the framework/paradigm is missing in those of games and entertainment artifacts. To fill the gap, in this article, by using games and entertainment artifacts as the source of inspiration, we put forward a novel systematic design process named GEA-inspired Design (GEAD) in the context of robotics in order to improve the cleaning performance in the way of endowing reconfigurability to cleaning robots and designing corresponding planning algorithms based on tiling theory. Similar with the bio-inspired counterpart, the GEAD typically follows two design threads. One is the problem-driven process where the problem arises from the field of robotics. The solution is inspired by GEA and solved based on principles extracted from existing solutions in the GEA domain, using a step-by-step approach. Another is the solution-driven process following a solution-to-problem pattern where the interesting GEA is determined to be an inspiration at the beginning and used to solve specific problems after transforming the GEA solution to the robotics domain.

## GEAD Paradigm

Although the concept and application of GEA-inspired design have been there for quite a period of time, the underlying paradigm has not yet been extracted and generalized. Furthermore, the process is usually dynamic and undergoes repetitive reformulations for both design problems and solutions. Therefore, the specific design changes frequently with the context of the problem faced and available solution spaces. Enlightened by the bio-inspired counterpart (Vattam et al., 2007; Helms et al., 2009), we propose here a GEA-inspired paradigm. In this section, we will be detailing both the problem-driven and solution-driven design threads. The problem-driven approach starts with an existing problem in real world and the solution-driven approach starts with an existing GEA solution.

### Problem-Driven GEAD Process

**Figure 2:**
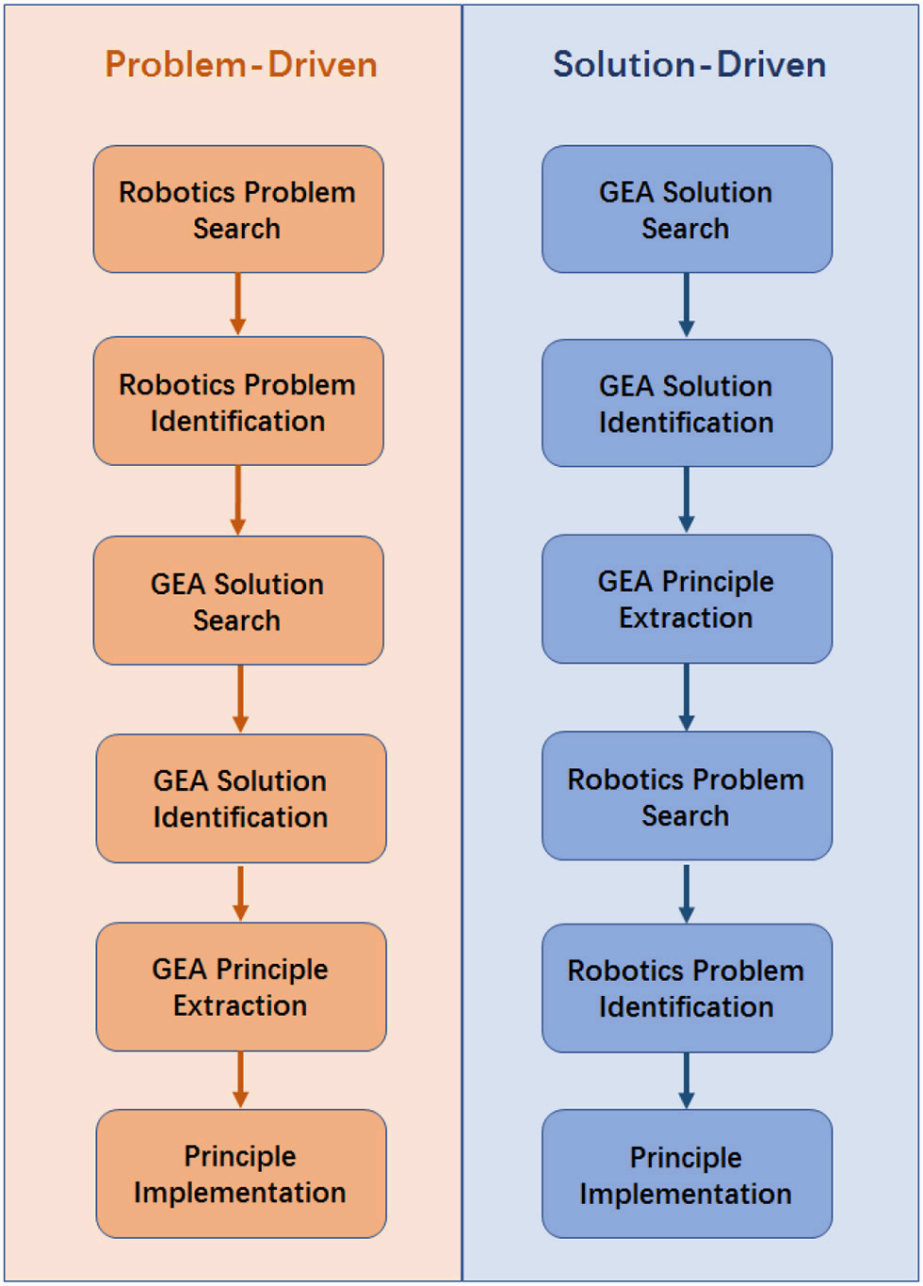
General GEAD processes for robotics, driven by problems and solutions respectively.

The problem-driven design process starts with an existing real-world problem, which follows the sequences of robotics problem search, robotics problem identification, GEA solution search, GEA solution identification, GEA principle extraction, and the principle implementation. To capture the general features of the problem-driven GEAD process, a diagram of the full process is presented in the left side of Figure 2 with short descriptions. Initially, the problem-driven process starts with the problem search and identification. In this step, an existing problem in real world is searched to be solved. Once the problem is founded, the deep understanding and interpretation are gone through. We then define a series of success criteria required to solve the problem. This involves the identification of multiple technical functionalities and reformulation of those functionalities into specific or abstract features. Given the required design criteria, we search the GEA solutions that satisfy the required features identified in the previous step. Candidates that meet parts of these features are shortlisted from the world of games and entertainment artifacts. Subsequently, the most suitable candidate is selected as the inspiration source. This step is done based on preliminary intuition, understanding and analysis of the fitness of between the candidate features and the problem specifics. Next, the principle extraction proceeds to explore the in-depth features of the selected candidate in the design point of view. Then, the fundamental principles are successfully extracted. Finally, the extracted principles are verified and implemented to solve the problems in the way of making either physical robots or running motion algorithms.

### Solution-Driven GEAD Process

Different from the problem-driven thread which is motivated by a problem in real applications, the GEAD process could be driven by the GEA solution. Such a solution-driven GEAD process is shown on the right-hand side of Figure 2, which follows the sequences of GEA solution search, GEA solution identification, GEA principle extraction, robotics problem search, robotics problem identification, and principle implementation. Initially, the GEA of interest are identified and listed down, which is typical done via observation, intuition, and inspiration. The selection of the GEA for inspiration is somehow subjective, intuitive, and mostly depends on the designer's perspective. Nevertheless, the GEA could be evaluated based on a few criteria such as but not limited to ease of principle extraction, ease of kinematic studies, and feasibility for practical implementation. Thus, the GEA inspiration source is selected based on these criteria case by case. Once selected, a deeper analysis is conducted based on its usage, feature, and various other aspects. As a result, a list of principles is extracted and gets reframed using engineering terminologies to fit in the robotics context. Given the extracted principles, a problem in the field of robotics (including the robot itself and applications of robotic techniques) is searched and identified, to which the extracted GEA-based solution applicable principle could be applied. Finally, the principles are applied and implemented to solve the identified problem in the previous step in the way of being a robotic prototype or an algorithm.

## Applications of GEAD Paradigm

**Figure 3:**
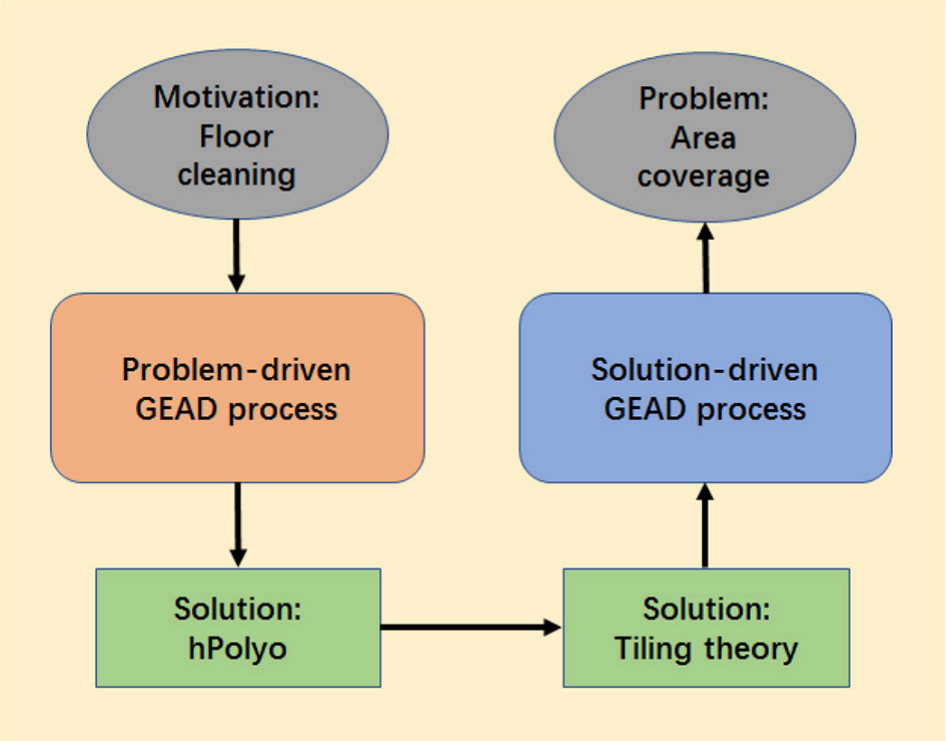
The schematic diagram of the GEAD process applied to floor cleaning in our case study.

Based on the proposed GEAD processes, the applications are presented in the context of domestic floor cleaning. The design of the cleaning robots and coverage algorithms are inspired by polyomino-relevant games and toys. The schematic diagram is shown in Figure 3 where two threads are involving: the left-hand side thread results in the robotic platforms following the problem-driven GEAD process and the right-hand side thread leads to the area-coverage strategy following the solution-driven GEAD process. As mentioned above, the GEA solution is typical from observation, intuition, and inspiration. From the diagram, the GEA-based inspiration could arise from the outcome of the previous GEAD process as well.

### Problem-Driven Process for Robot Design

Here we follow the problem-driven GEAD process to create a series of robots called hPolyo which are inspired by the polyomino-relevant games and toys. The robots are capable of switching between different forms of polyominoes, according to the perceived environment with an objective of maximizing the floor area coverage.

P1) Robotics Problem Search. Firstly, we look for the potential problems that can be solved by robots. As one of the most common application of robots, floor cleaning is the focus of this article.

P2) Robotics Problem Identification. One of the significant challenges affecting the performance of floor cleaning robots is the difficulty in cleaning spaces of room corners, narrow corridors and in between furniture. This leads to the inability to cover the maximum area. Moreover, the design of the robot should consider area coverage in both small and large areas. Currently, the floor-cleaning robots have their limitations in cleaning underneath furniture and accessing narrow corridors, which are mostly due to robot's size and shape.

The problem can be reframed as the following criteria:

- The robot should be relatively small in order to navigate through narrow openings.
- The robot should be able to change morphologies while adapting to the geometries of rooms and the furniture.
- The robot should be able to team with other robot in order to clean a large area collaboratively given the timing limitation. The collaboration issue has not been considered in the design of currently available cleaning robots which are basically for home applications. Simply deploying multiple robots may cause issues like redundant cleaning due to trajectory overlap, collision, etc.

**Figure 4:**
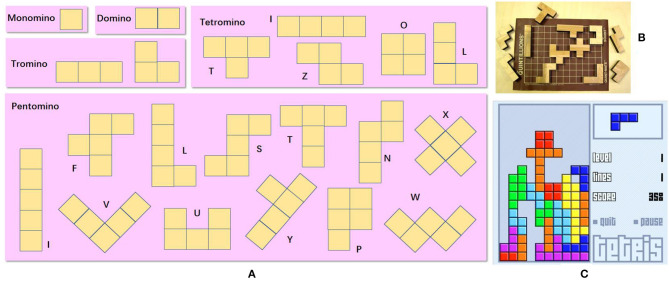
**(A)** Different types of polyominoes (Lo et al., 2009). **(B)** Commercial product Quintillion, courtesy of Kadon Enterprises, Inc. **(C)** Tetris, courtesy of Tetris Holding.

P3) GEA Solution Search & Identification. The GEA solutions related to the change of morphology and fitting piece into different gaps are searched. As a result, we have a list of puzzle matching games. The polyomino puzzles involve the manipulation of modules with different shapes (Figure 4A). Such a kind of GEA (Figures 4B,C) are chosen because they are composed of regular squares/cubes which make it easier to implement physically. Among these polyomino puzzles, Tetris is one of the most famous tile-matching video games, which evidently satisfies the second and third criteria in P2).

P4) GEA Principle Extraction. Different polyomino puzzles share many same characteristics. They are based on polyominoes whose geometric configurations are composed of four squares connected orthogonally. Specifically, Tetris can turn into seven distinct tetrominoes, where three of them are reflectional symmetry and the rest exhibit chirality where their mirror images are not identical. The principles extracted from Tetris are as follows:

- A single module is made of four equally sized interconnected blocks.
- The rearrangement of the blocks can produce different shapes.
- In Tetris, the shape of the module is decided based on the required scenario of the game by the player. In other words, the shape of the module is decided based on which area the module is supposed to be fitted on to.
- Tetris exhibits intra-reconfiguration where a single module can change its morphology without requiring an external assembly or disassembly of blocks.
- Tetris exhibits inter-reconfiguration where multiple modules join to form a new configuration.

Essentially, the tetromino is one type of polyomino, and thus the extracted principles can be extended to any types of robots inspired by polyominos, which are called hPolyo.

**Figure 5:**
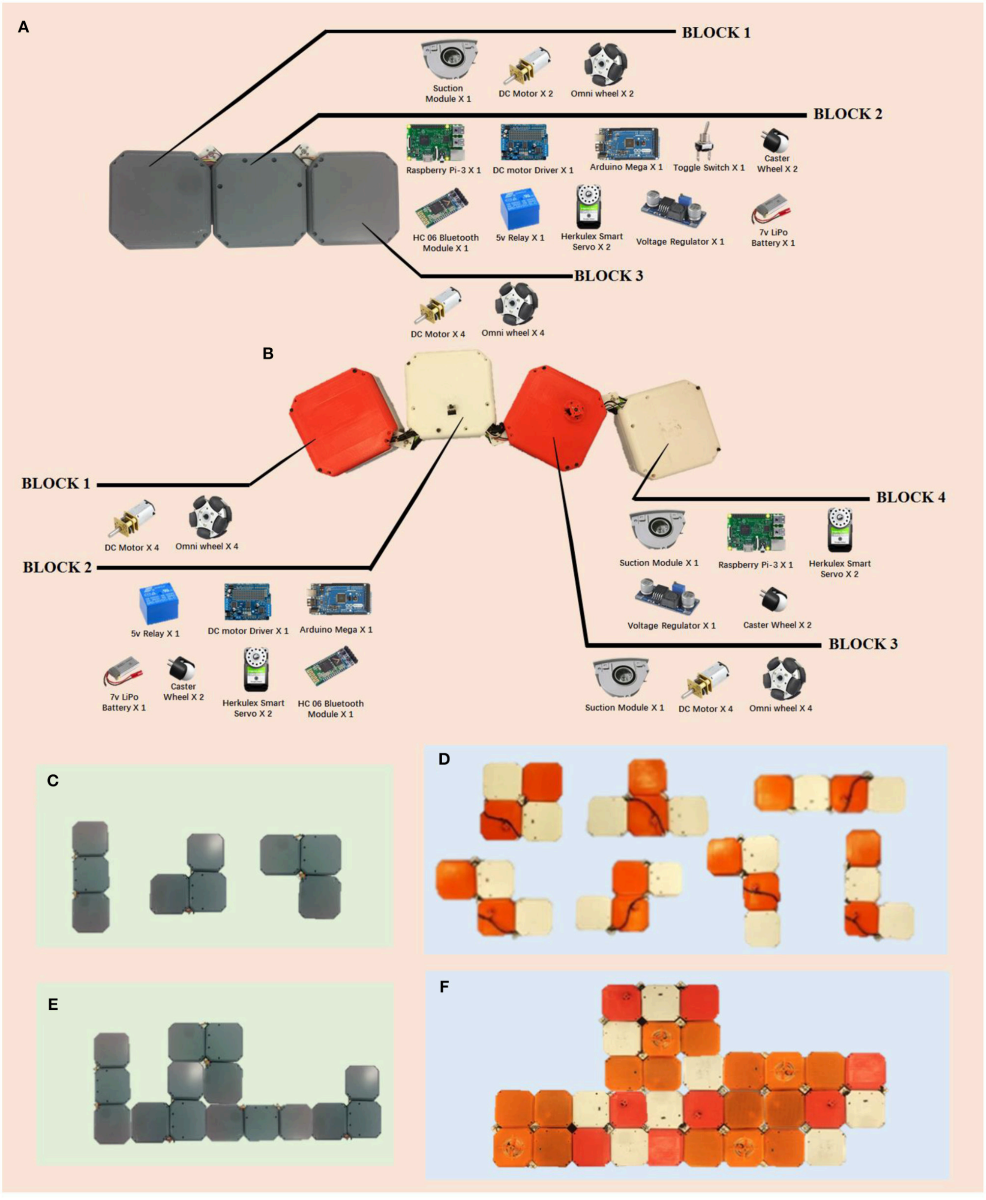
The two types of hPolyo and their component lists. **(A)** The prototype of hTromo robot inspired by trominos. **(B)** The prototype of hTetro robot inspired by tetrominos. **(C)** Three morphologies of hTromo. **(D)** Seven morphologies of hTetro. **(E)** Assembly of multiple hTromos. **(F)** Assembly of multiple hTetros.

P5) Principle Implementation. Here we develop two types of hPolyo: one design is inspired by trominos called hTromo (Figure 5A) and another design is inspired by tetrominos called hTetro (Figure 5B). The extracted principles from polyomino puzzles are implemented into hTromo and hTetro as depicted as follows:

- The structural designs of hTromo and hTetro are modular, forming by four size-equal hinged-connected blocks.
- Similar to the shape-shifting in Tetris, the two designs exhibit intra-reconfiguration where one single robot can reconfigure its internal morphology without requiring any external assembly or disassembly (Figures 5C,D). This endows the robot the increased cleaning performance when navigating around the internal contour of the room such as narrow corridors and underneath furniture.
- Similar to the tile-matching in polyomino puzzles, a collection of robots exhibits inter-reconfigurability where individual robots can dock with others as shown in Figures 5E,F. This principle signifies that the combination and collaboration of multiple robots could cover larger areas.

The physical prototypes of hTromo and hTetro are made of four blocks connected by three active hinged dissections. These three active hinges help the robot in transforming between the seven morphologies. Each block is designed based on specific design requirements. Block 1 is responsible for the robot's overall mobility, Block 2 houses the controllers and electronic modules, Block 3 and 4 are reserved to dwellings in the cleaning apparatus. Each block of hTromo measures 140 × 140 × 75 mm, and each block of hTetro has a dimension of 140 × 140 × 55 mm. More details about the specific design of the robot can be referred to Prabakaran et al. (2017, 2018).

**Figure 6:**
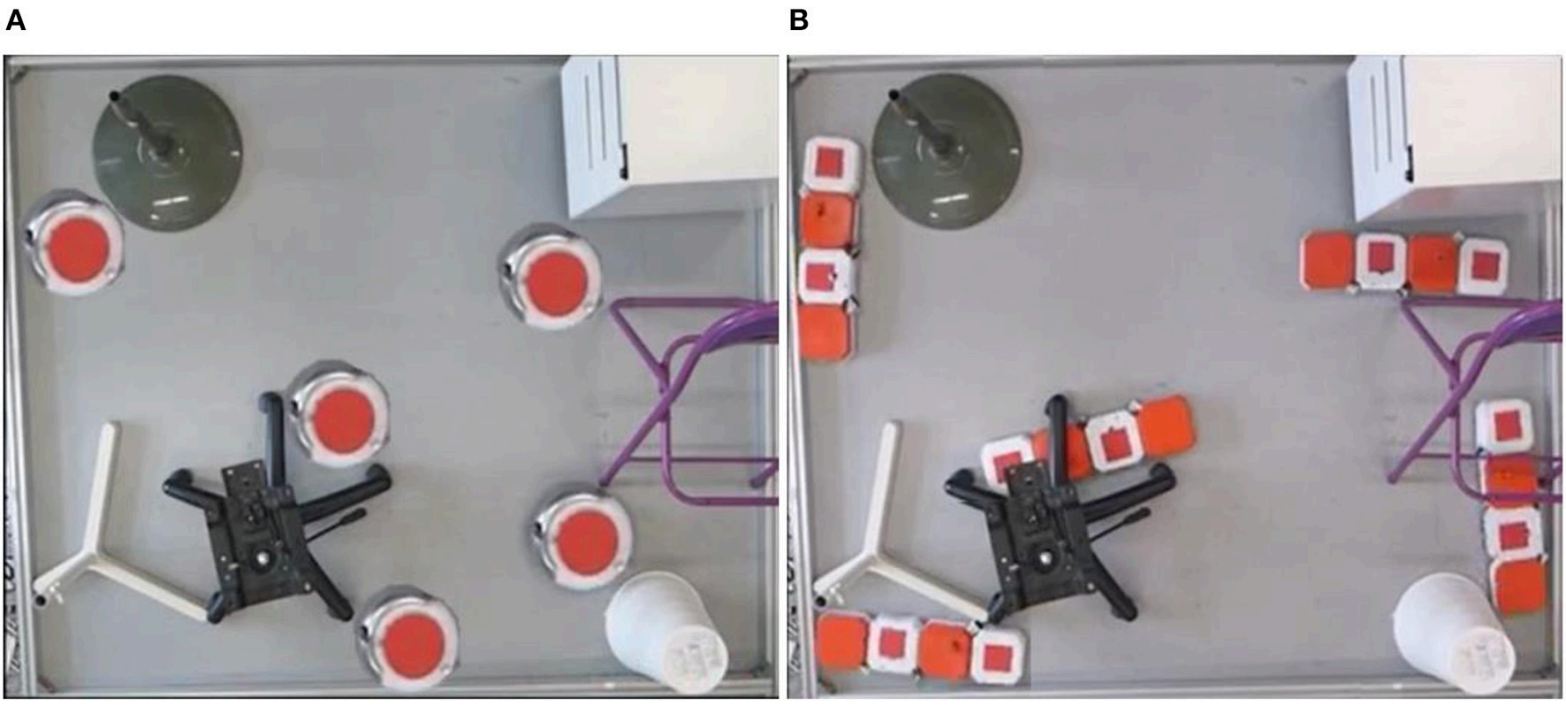
Comparisons of conventional floor cleaning robots and hTetro in space access and area coverage. **(A)** The conventional floor cleaning robots cannot enter the areas between the furniture and the wall. **(B)** hTetro can enter the areas that the conventional robot cannot.

Generally speaking, hTromo and hTetro are polyomino-inspired reconfigurable robots which can tackle both small and large area cleaning tasks via nested reconfiguration (Tan et al., 2015). The intra-reconfigurability of the robot allows the robot to navigate through small areas, and the inter-reconfigurability boosts to cover large area rapidly. Figure 6 demonstrates the comparison of cleaning scenarios between traditional cleaning robots with circular shape and hTetro with smaller cross section. Table 1 gives the comparison results of the two robots for accessing six typical areas. While facing the same conditions, hTetro can enter all these narrow areas because the I-shape is thin enough to fit in those areas, whereas the traditional cleaning robot is not able to navigate into those areas because of oversize. These comparative experiments show that our shape-shifting robot is more efficient than the shape-fixed robot in terms of space access and area coverage.

**Table 1:** Experimental results of comparisons of traditional cleaning robot and reconfigurable cleaning robot (red arrows indicate the access point/area; 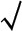 indicates the successful access; 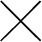 indicates the failed access).

	**Access point/area**					
**Cleaning Robot**	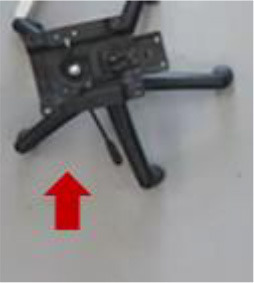	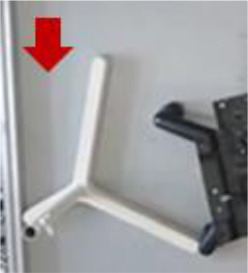	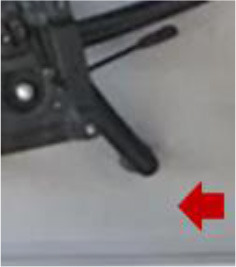	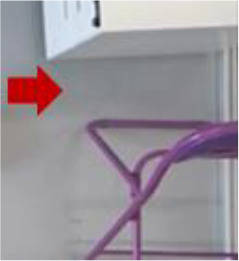	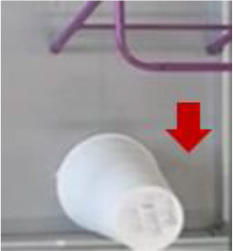	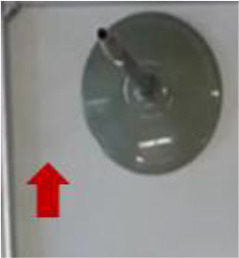
Traditional design	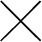	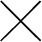	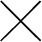	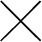	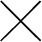	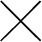
Proposed design	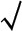	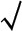	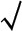	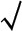	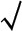	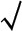

**Figure 7:**
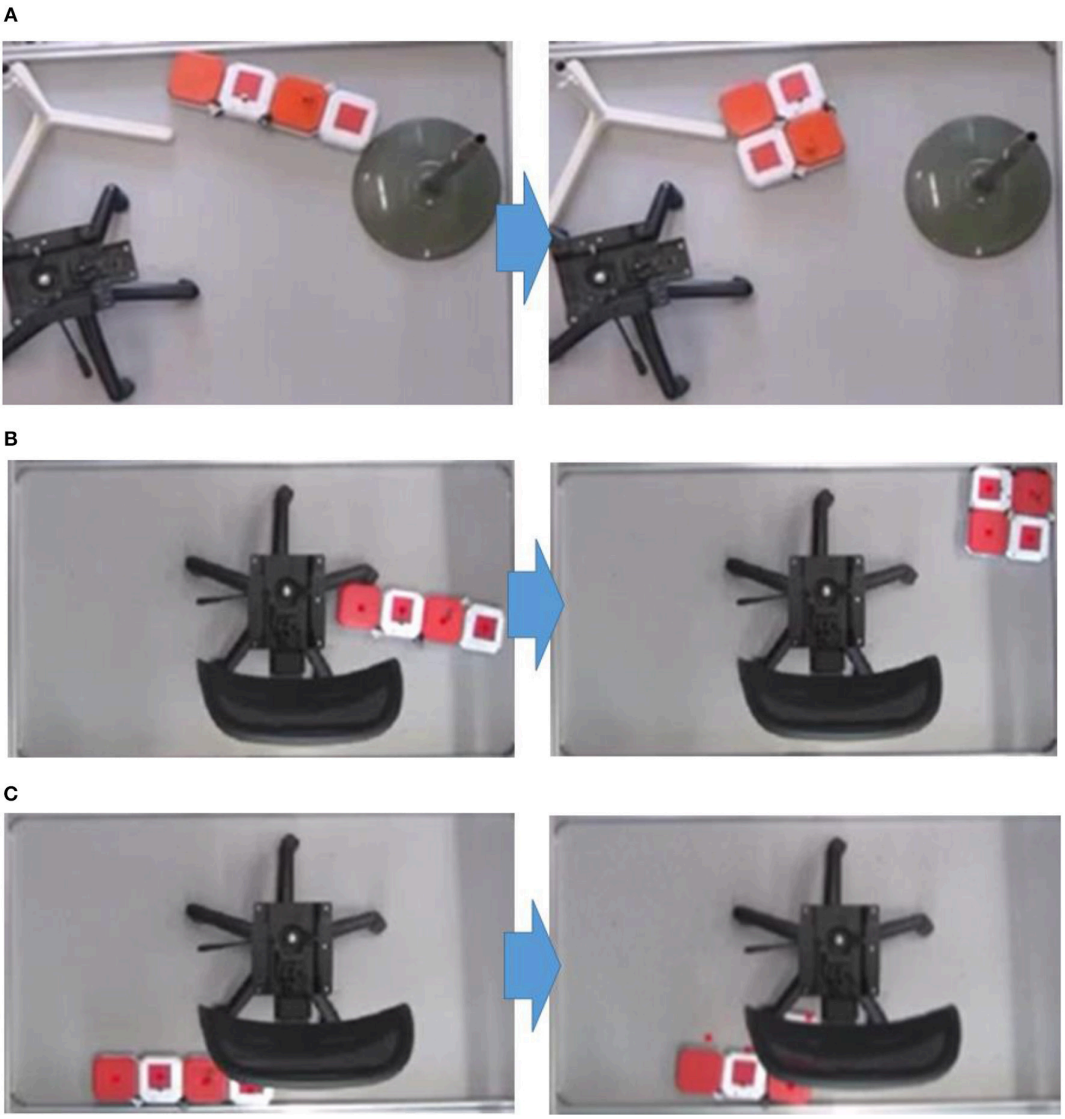
Once the initial configuration is not fit for crossing the opening, hTetro can shift to an appropriate shape through reconfiguration. **(A)** Reconfiguration from I-shape to O-shape. **(B)** Reconfiguration from I-shape to O-shape. **(C)** Reconfiguration from I-shape to L-shape.

Moreover, from Figures 7A,B, we can see that hTetro can leverage the intra-reconfiguration to assist navigation subjected to space constraints. Once the initial configuration is not fit for crossing the opening, hTetro can shift to an appropriate shape through reconfiguring from I-shape to O-shape. Figure 7C demonstrates that the intra-reconfiguration enables hTetro to clean by shifting from I-shape to L-shape while moving through the narrow space between the chair and wall. It shows that the reconfigurability makes the cleaning more efficient. The video of the experiments can be referred to^1^.

The above experiments showcase the superior capability of single modules of hTetro. As mentioned before, multiple modules of hTetro could cover larger areas. This means there are potential applications in public-area cleaning, such as car park, shopping mall, etc.

### Solution-Driven Process for Area Coverage Strategy

Here, we follow the solution-driven GEAD process to design the strategies to solve the area-coverage problem in floor cleaning tasks.

S1) GEA Solution Search & Identification. In polyomino puzzles, it basically involves two-dimensional tiling problems in which a number of polyomino blocks have to be assembled into a larger given shape without overlaps and gaps. We consider the tiling problem as a GEA solution to explore.

S2) GEA Principle Extraction. Usually, there are more than one tiling strategies that can solve a tiling problem. The polyomino tiling process is governed by tiling theorems. The tiling theorems deal with the problem of partitioning or filling of a geometrical region using same or multiple sub-regions. Literature offers numerous work that discusses tiling theorems with proof for distinct polyomino sets. Although there has been a lot of research work involving applications of polyomino tiling theories. However, very few works involving tiling theory has been applied in the field of robotics research and robotics related engineering applications.

Thus, the principle behind the tiling puzzles is the tiling theory that formulates the tiling rules in a mathematical way. So far, there have been numerous theorems in mathematics that can potentially describe and interpret the polyomino tiling problems. Four theorem examples are given as follows where Theorem 1 is for trominoes and the rest are for tetrominoes.

Theorem 1 Golomb (1996): An *a* × *b* rectangle can be tiled with L- and I- trominoes if and only if that rectangle is divisible by 3.

**Figure 8:**
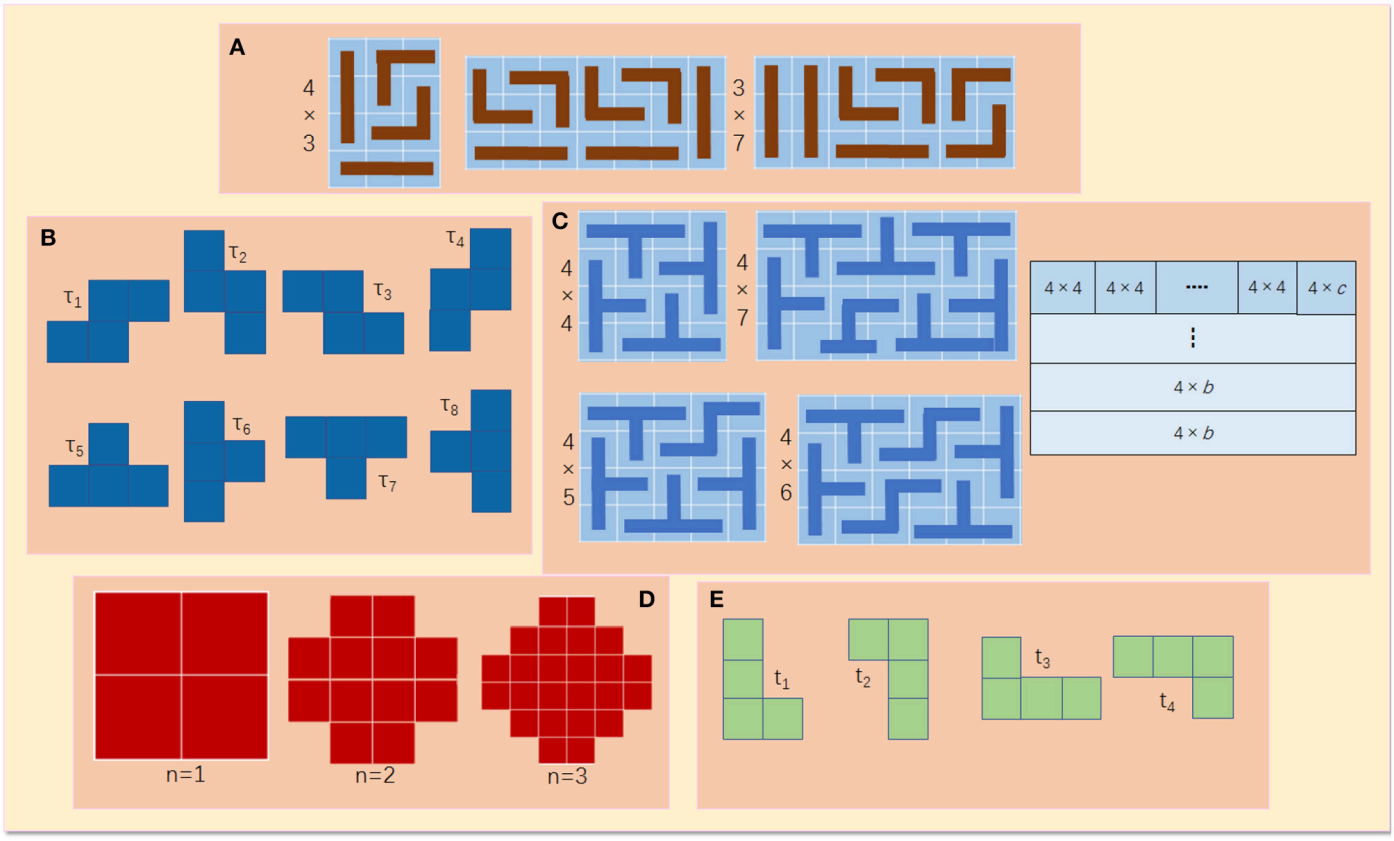
**(A)** Tiling examples of 4 × 3 and 3 ♢ 7 rectangular areas. **(B)** Set *Σ* of tetrominoes. **(C)** Image argument for Theorem 2. **(D)** Aztec diamond of order 1, 2, and 3. **(E)** Set *Ψ* of trominoes.

Figure 8A shows three examples of tiling 4 × 3 and 3 × 7 rectangular areas using L- and I-trominoes.

Theorem 2 (Lester, no date): An *a* × *b* rectangle can be tiled by the set *Σ* if, and only if, *a, b* ≥ 4 and *a* or *b* is divisible by 4.

Σ = {τ_1_, …, τ_8_} is a set of tetrominoes including of four skew tetrominoes (τ_1_, …, τ_4_) and four T-tetrominoes (τ_5_, …, τ_8_). Since the tetromino has four constituent squares with unit area, any rectangle with an area that is a multiple of 4 can be covered using any of the tetrominoes. Let *a* and *b* be the dimensions of the rectangle to be tiled. Since the area of the rectangle has to be a multiple of 4, (*a* × *b*) = 4*n*, where *n*∈ {1, 2, 3…}. This implies that either *a* or *b* must be divisible by 4. Let *a* = 4 and 4 ≤ *b* ≤ 7, then a 4 × *b* rectangle can be tiled using the arrangement of skew and T-Tetrominoes shown in Figure 8B. Let *b* > 7 and *c* ∈ {4, 5, 6, 7}, then *b* = 4*n*+*c*, where *n* can have a positive integer value. As such, it allows for splitting of a (4 × *b*) rectangle into *n* (4 × 4) rectangles and one (4 × *c*) rectangle, as in Figure 8C. This implies, if b ≥ 4, then a (4 × b) rectangle can be tiled using a set of T- and skew tetrominoes. In the case of *a* = 4*m*, where m is a positive integer, then a rectangle (*a* × *b*) can be decomposed into *m* (4 × *b*) rectangles as shown in Figure 7D. Therefore, an (*a* × *b*) rectangle can be tiled using T- and skew tetrominoes.

Theorem 3 Lester (2012): Let a and b be integers that are strictly greater than 1. As such, Set Ω of tetrominoes can tile a modified rectangle of side a and b, if, and only if, either:

- *a* ≡ *2 (mod4) and b is odd, or*
- *b* = *2 and a* ≡ *1 (mod 4)*.

Ω = {τ_1_, τ_3_, τ_6_, τ_8_} is formed of a pair of T-tetrominoes and skew tetrominoes. The term *modified rectangle* in the theorem describes a rectangle of sides *a* and *b* with a single square truncated from both corners. As per Theorem 2, four must divide the area of the modified rectangle for the area to be completely tiled using tetrominoes. Hence, it is clear that (*ab*−2) must be divisible by four.

Theorem 4 Chao et al. (2013): A k-inflated Aztec diamond of order n can be tiled by Set *Ψ* if and only if k is even. If the tiling is possible, then it follows the domino pattern.

The *k*-inflation of a given polyomino is defined to be a larger copy of the initial polyomino in which all 1 × 1 squares are replaced by *k* × *k* squares. Figure 8D shows the Aztec diamonds of order 1, 2, and 3, respectively. Set Ψ is given in Figure 8E.

These theorems will be used to exemplify the applications of tiling theory through simulation/ animation and experiments in the principle-implementation step.

S3) Robotics Problem Search. There is no prior research work that had been done in applying the tiling theory in robotics field. Whereas, we realize that the tiling rules that are determined by tiling theorems can be used as the planning strategy for area-coverage problems, which are often encountered in deployment of service robots.

S4) Robotics Problem Identification. The area coverage is a fundamental problem in floor cleaning scenarios. There has no algorithm designed for the reconfigurable robots, specifically, for the hPolyo robots developed in this case. Thus, we decide to apply the tiling theorems presented in S2) to designing area-coverage strategies (i.e., tiling sets) for hTromo and hTetro.

**Figure 9:**
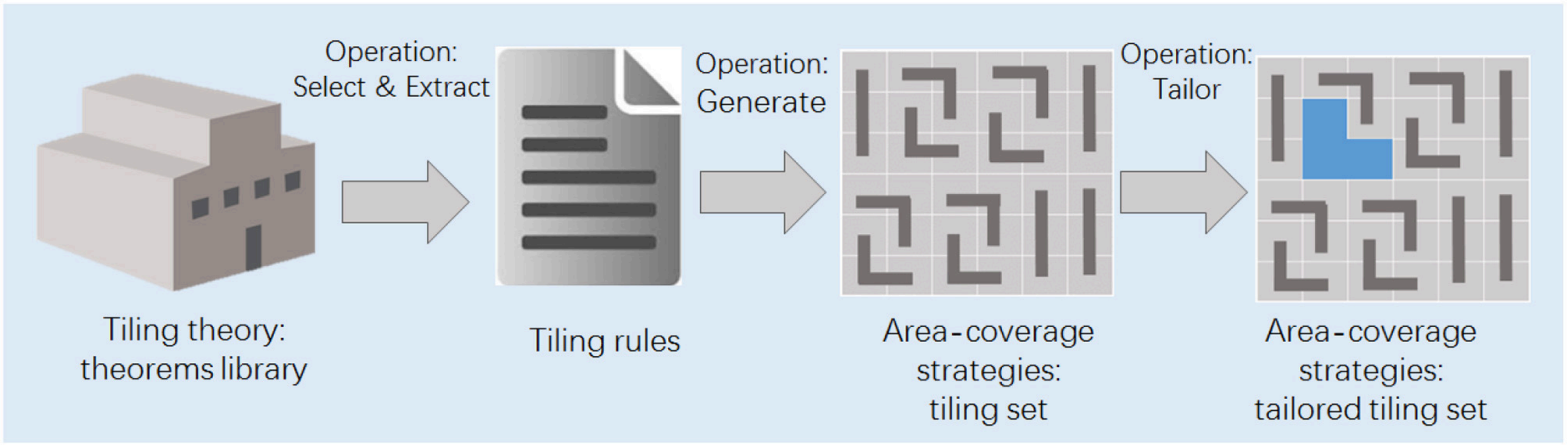
The schematic diagram showing the transfer process from tiling theorems to area coverage strategies.

S5) Principle Implementation. After extracting the principle and identifying the problem, we proceed to implement the principle into robotic applications. The process of transfer from tiling theorems to area coverage strategies is illustrated in Figure 9. First, the tiling theorems from the world of tiling theory are selected and extracted into tiling rules. Based on the tiling rules, the tiling sets are generated. Then the tiling sets are organized as groups of configurations are determined based on the rules. Our platform can generate the global tiling set required to cover a given area by leveraging the polyomino tiling theorems autonomously.

**Figure 10:**
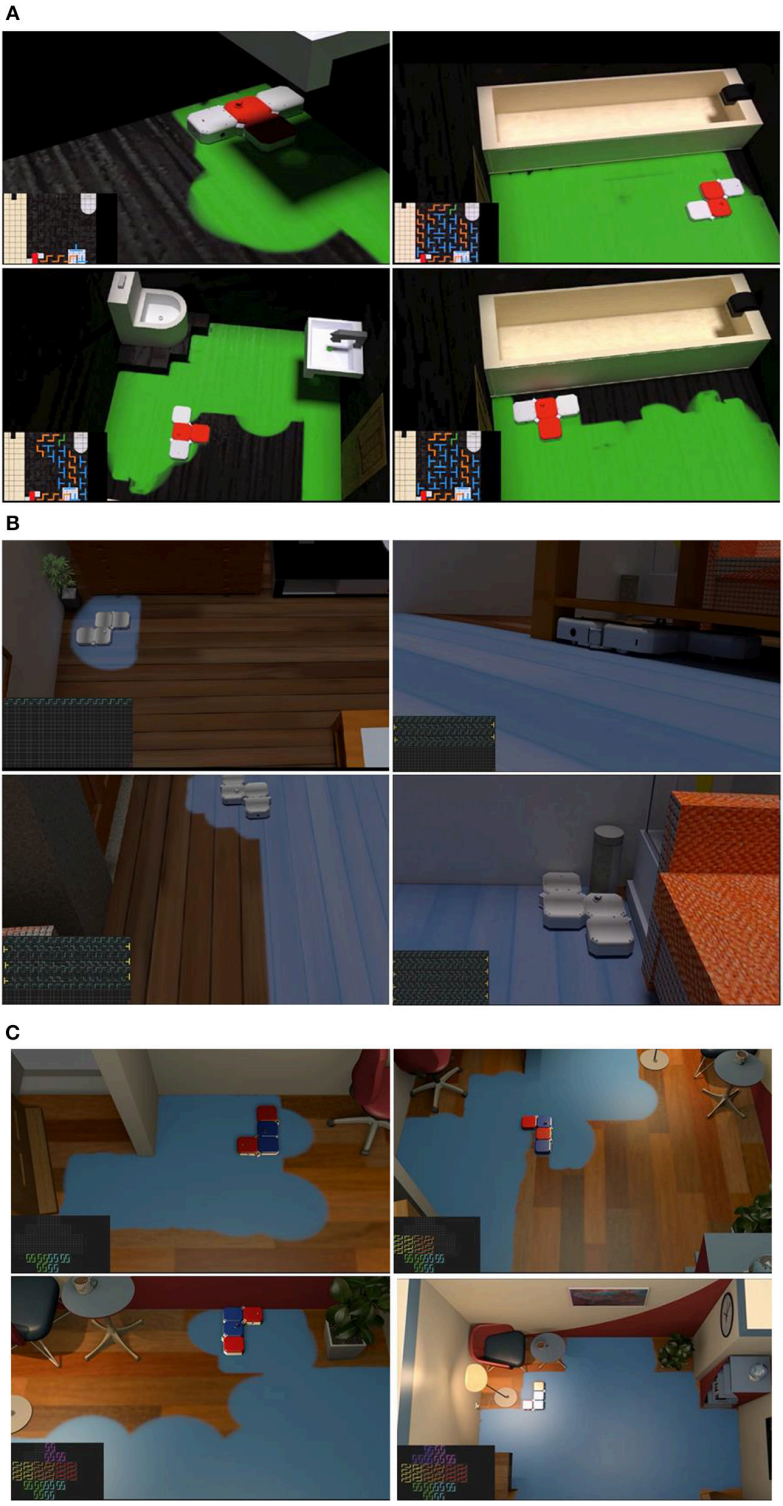
Simulation/Animation for implementing the area coverage strategy with hTetro, based on tiling theorems. **(A)** Bathroom scenario with strategy based on Theorem 2. **(B)** Living-room scenario with strategy based on Theorem 3. **(C)** Study scenario with strategy based on Theorem 4.

Figure 10 demonstrates the simulation animation for implementing the area coverage strategy with hTetro. Theorem 2, 3, and 4 are applied to generate tiling sets to cover the bathroom (Figure 10A), living room (Figure 10B), and study scenario (Figure 10C), respectively. The tiling sets are fitted in the specific environments where corresponding configurations are removed from the planning strategy when there are other settings such as closes tool, bathtub, and furniture. These tailored tiling sets guide the reconfigurable robot to reach where it can. The full versions of animations are referred to the videos accompanying this article, which show that hTetro can fulfill the task of room-floor coverage.

**Figure 11:**
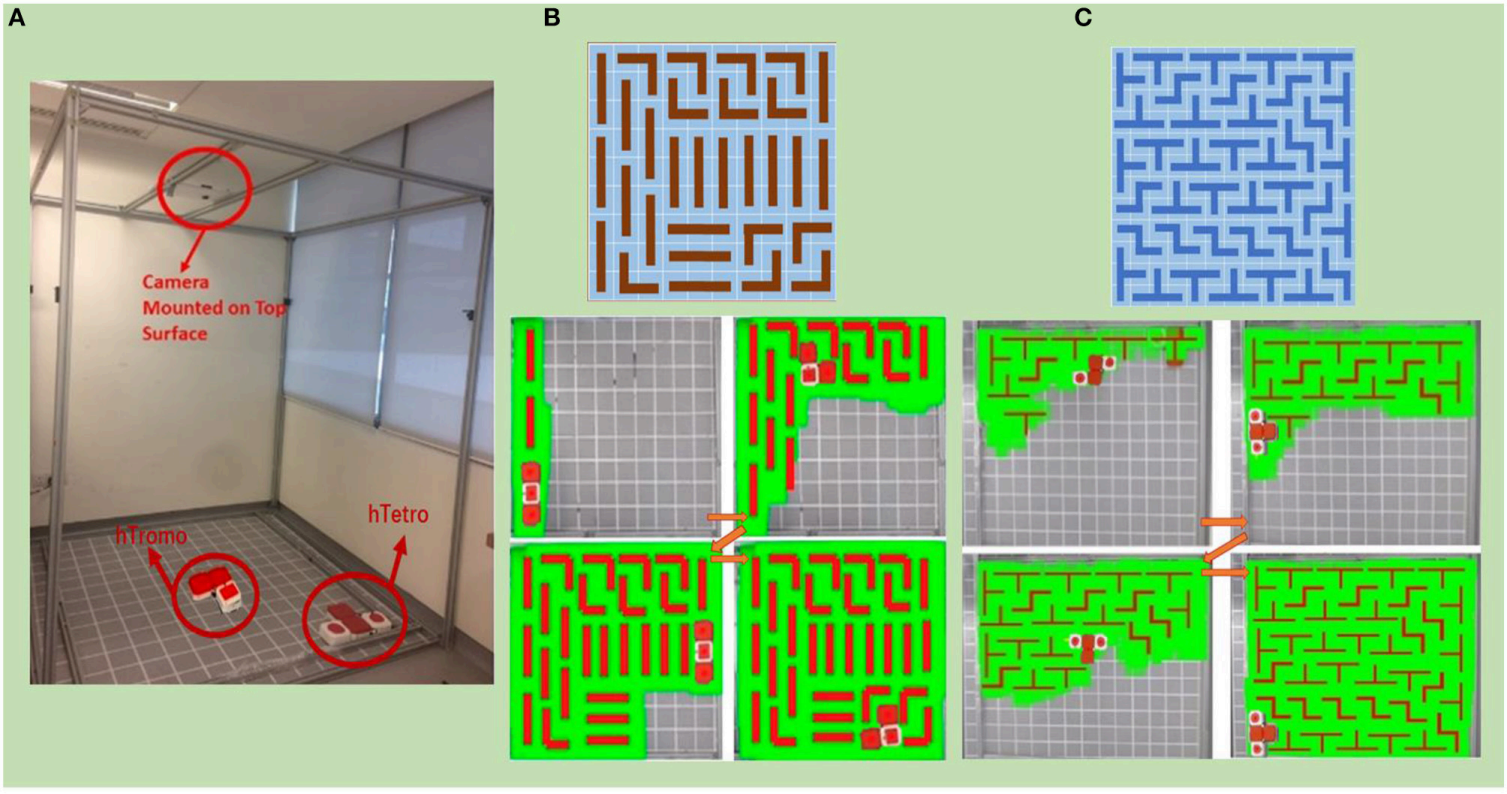
**(A)** Experimental tested for implementing the area coverage strategy based on tiling theory. **(B)** Experiments validating application of Theorem 1 in hTromo. **(C)** Experiments validating application of Theorem 2 in hTetro.

Experiments are conducted as well within an area of 196 cm× 196 cm with 14 × 14 grids as shown in Figure 11A. An image-acquisition device attached to the overhead support frame is deployed to benchmark the cleaning performance in term of coverage ratio. The percentage of coverage area calculation is conducted using the following equation

(1) $$\text{Coverage ratio}=\frac{\text{Pixels of the robot occupied}}{\text{Total pixels of the testing area}}\;\times \text{100}\%$$

to assess the total area coverage achieved. Based on Theorem 1, hTromo follows a set of configurations to cover a 140 × 126 cm area split into 10×9 grids, as shown in Figure 11B. A rectangular plot of 168 × 182 cm segmented into 12 × 13 squares is used to validate Theorem 2 using hTetro. Figure 11C presents the tiling set and track map images of the tiling process. In both Figures 11B,C, the green-colored shading represents the area covered by the robots. The experimental results indicate that the hTromo robot achieves a coverage rate of 95.5%. Similarly, the hTetro robot achieves a coverage rate of about 95%. The residual areas that have not been covered distribute around the boundaries of the test site, which are due to the motion uncertainties of the robots.

## Conclusion

In this article, we put forward a general design paradigm called GEAD process in the context of robotics-related applications. Similar to the bio-inspired counterpart, we discussed two distinct threads of GEAD, which are namely problem-driven and solution-driven, respectively. Based on the proposed design process, we have managed to successfully prototype two reconfigurable floor-cleaning robots named hTromo and hTetro, respectively. The robots were inspired by polyomino puzzle games and toys. The robots demonstrated inter- and intra-reconfiguration capabilities. The cleaning performance of the proposed robots are compared with that of the conventional floor-cleaning robot with experimental validations, which evidenced that our robots are capable of overcoming these limitations thanks to their reconfiguration capabilities. Moreover, we have implemented the polyomino tiling theory to develop a novel area-coverage strategy. This strategy is fitted in with the developed reconfigurable robots which leverages polyomino tiling sets to cover the given areas.

We have validated the proposed strategy using simulations and experiments in different scenarios. The experiments validated that the intra-reconfigurability improve the cleaning. The comparison between traditional cleaning robots and hTetro demonstrated that hTetro can enter all these narrow areas because the thin I-shape can fit into those areas, whereas the traditional cleaning robot is not able to navigate into those areas because of oversize. The comparative experiments showed that our shape-shifting robot is more efficient than the shape-fixed robot in terms of space access and area coverage. Moreover, hTetro could leverage the intra-reconfiguration to navigate through constrained openings through reconfiguration. Besides, the modularity enables that multiple modules of hTetro could cover larger areas. This means there are potential applications in public-area cleaning, such as car park, shopping mall, etc. Both simulations and experiments demonstrated that hTromo and hTetro can cover different types of scenes with different layouts, such as bathroom, living room, and study scenario. This means that the tiling strategies are quite suitable to this kind of specially designed robots.

It is worth mentioning that the proposed paradigm a general framework. In most cases, the original designers of the robots might not follow the same process strictly when designing the robots. Actually, the proposed paradigm is flexible that one or some of steps could be missing in real implementation. However, this paradigm could guide the robot designers to converge the design plans step by step. Therefore, the paradigm can standardize the process of robot design.

## Author Contributions

NT researched the literature, substantially wrote the paper, and contributed to the analysis of results. NB drafted part of work. RM initiated and designed the work. VP contributed to the development of prototypes and experiments. All the authors approved the submitted version of the manuscript.

### Conflict of Interest Statement

The authors declare that the research was conducted in the absence of any commercial or financial relationships that could be construed as a potential conflict of interest.

## Footnotes

^1^*hTetro: Shape Shifting Floor Cleaning Robot - YouTube*, 2018.
